# Health Benefits of a Plant-Based Dietary Pattern and Implementation in Healthcare and Clinical Practice

**DOI:** 10.1177/15598276241237766

**Published:** 2024-03-14

**Authors:** Matthew J. Landry, Catherine P. Ward

**Affiliations:** 1 Department of Population Health and Disease Prevention, Program in Public Health, 8788 University of California, Irvine, CA, USA (ML); 2 Stanford Prevention Research Center, School of Medicine, Palo Alto, CA, USA (CW)

**Keywords:** plant-based, vegetarian, vegan, nutrition counseling, lifestyle medicine

## Abstract

The American College of Lifestyle Medicine recommends eating a predominantly plant-based diet with a variety of minimally processed vegetables, fruits, whole grains, legumes, nuts and seeds. At any level, adoption of a plant-based diet can improve one’s health through a variety of mechanisms. Increasing intake of plant-based foods often results in increases in fiber intake, decreases in saturated fat intake, and increased intake of essential vitamins and minerals, among other healthful benefits. Despite such potential benefits, many individuals are reluctant or resistant to change their usual dietary behaviors or unable to sustain diet changes over time. This is largely because an individual’s decision to adopt a plant-based diet is influenced by a diverse array of motivating factors, priorities, and/or misconceptions about nutrient adequacy of plant-based diets. Here, we discuss key points from a session at the American College of Lifestyle Medicine’s annual conference LM2023. Specifically, we review common preconceptions about plant-based diets, provide guidance on removing the barriers to adopting and adhering to plant-based diets, and highlight key literature findings supporting the health benefits of plant-based diets. Last, we discuss how plant-based diets are increasingly being implemented within health care and clinical practice to support Food is/as Medicine approaches.

“Consulting with a Registered Dietitian Nutritionist (RDs or RDNs) or a health care professional can provide personalized guidance to address individual nutritional needs.”

## Continuum of Plant-Based Dietary Patterns

Two of the most significant global health crises affecting society are climate change and the burden of noncommunicable diseases, which are both inextricably linked to diet.^1^ Despite contradictory media headlines, there is strong published evidence that dietary patterns high in plant foods and low in animal foods can maximize health, environmental, and economic benefits.^2,3^ Plant-based diets consist of a diverse family of dietary patterns, defined by a reduced intake of animal-derived foods.^4^ Table 1 shows the continuum of different dietary patterns which could all fall under the definition of being plant-based and the food groups they include or exclude. On one side of the continuum, flexitarian or semi-vegetarian diets are primarily plant-based but allow some animal-derived food consumption. On the opposing end are vegan diets which exclude all foods and beverages wholly or partly derived from animals. Even within these broad categories of plant-based dietary patterns, there are patterns such as the whole-food vegan diet which excludes processed foods. A commonality of all plant-based dietary patterns is inclusion of vegetables and fruits, legumes, whole grains, nuts and seeds.

**Table 1.** Variety of Plant-Based Dietary Patterns.

Type	Red Meat	Poultry	Fish and Seafood	Eggs	Milk and Dairy Products	Grains, Legumes, Nuts, Vegetables, and Fruit
Flexitarian (Semi-Vegetarian)^a^	X	X	X	X	X	X
Pollo-Vegetarian		X		X	X	X
Pescatarian			X	X	X	X
Lacto-Vegetarian					X	X
Ovo-vegetarian				X		X
Lacto-Ovo-Vegetarian				X	X	X
Vegan						X
Whole-Food Vegan^b^						X

^a^Primarily includes plant foods, occasionally includes animal-based foods.

^b^Excludes all animal and animal-derived products and processed foods.

## Broad Support of Plant-Based Diets from Professional and Medical Organizations and Societies

In recent years, there has been a paradigm shift in the perspective towards recognizing the significant impact of food and dietary choices on health outcomes. Increasingly, professional medical organizations are endorsing the idea that food can be a powerful form of medicine (i.e., Food is/as Medicine).^5^ In particular, numerous professional societies and organizations have published stances, positions, or clinical practice guidelines that recommend plant-based dietary patterns for preventing and treating certain diseases. The American College of Lifestyle Medicine’s dietary position statement recommends eating a predominately plant-based diet with a variety of minimally processed vegetables, fruits, whole grains, legumes, nuts and seeds.^6^ Other prominent organizations making this recommendation include the American Cancer Society,^7^ Academy of Nutrition and Dietetics,^8^ the American Heart Association,^9,10^ and the American College of Cardiology.^11^ Additionally, the Dietary Guidelines for Americans, 2020-2025^12^ includes a healthy, vegetarian-style dietary pattern that can be adopted for improved health and chronic disease prevention.

## Evaluating Plant-Based Research

Considering the expanding body of scientific literature on plant-based diets, practitioners must assess several factors when appraising the evolving evidence. Specifically, when scrutinizing studies comparing a plant-based diet to a comparator diet (e.g., Diet A vs Diet B) or investigating the association between a plant-based diet and a specific health outcome, we recommend that practitioners focus on 3 essential considerations: definition, design, and adherence.^13^

First, when reviewing studies, it’s important to know how researchers are defining a plant-based diet. Table 1 illustrates the range of plant-based diets that may all fall under the same umbrella of plant-based. For example, two studies both testing the effects of a vegetarian diet might have varying definitions for foods that are included/excluded within a vegetarian diet. A second key element is considering study design, in which participants are randomized to a plant-based diet or a comparator diet or is it a longitudinal study that compares diets over a period of time. Perhaps it is a cohort study for which a person’s dietary habits are either examined retrospectively or prospectively. Additionally, within these study designs, it’s important to examine the length of time participants remain on a plant-based diet: 3 months, 3 years, or 3 decades? Last, a major factor for consideration is adherence of study participants over time: do participants strictly follow a plant-based diet or occasionally have “cheat days” or situations where they may consume some animal products?

Much of the existing evidence in support of plant-based diets has been gleaned through observational epidemiologic studies, particularly prospective cohort studies, which focused on following cohorts over time, collecting data on their exposure to the diet.^14^ Two notable examples are the Oxford cohort of the European Prospective Investigation into Cancer and Nutrition (EPIC) study (EPIC-Oxford) and studies of Seventh-day Adventists in the US culminating in the Adventist Health Study 1 and 2.^14^ Numerous published randomized controlled trials (RCTs) have provided valuable insights into the health benefits of a plant-based diet compared to an omnivorous diet. Other RCTs have delved into the distinctions between different types of plant-based diets (e.g., vegetarian vs vegan) for their respective impacts on health. Both study designs (observational and RCTs) are critical for us to understand the role plant-based diets play in health outcomes because they often answer different research questions in different study populations (e.g., general compared with high-risk population) and may differ in focus (e.g., primary compared with secondary prevention).

## Plant-Based Components and Benefits in Prevention and Treatment of Chronic Disease

Plant-based diets are typically low in saturated fats and dietary cholesterol while providing health-promoting foods rich in fiber, antioxidants, and phytochemicals. These dietary attributes partially explain how plant-based dietary patterns may be associated with or result in positive health attributes that are observed in both observational studies and randomized controlled interventions.^15^ The evidence linking plant-based diets to cardiovascular disease prevention is particularly robust.^16^ Plant-based diets are associated with decreases in cardiovascular disease risk factors such as improved weight,^17-19^ lipid management,^15,17,20^ glucose metabolism,^18,21^ and blood pressure.^22^ Additionally, plant-based diets are associated with lower cardiovascular disease incidence^15,23-25^ and cardiovascular disease related mortality.^15,24,26,27^ Recent studies of plant-based diets have also observed a lower risk for type 2 diabetes^15,25^; a decreased likelihood of various types of cancer including prostate, colorectal, breast, and digestive system ^25,28-31^; and improved prognosis among cancer survivors.^28,32^

## Effects of Plant-Based (Vegan) Diet vs Omnivorous Diets Among Identical Twins

Although it’s well-known that eating less meat improves cardiovascular health as we have discussed, dietary interventions are often hampered by factors such as genetic differences, upbringing, and lifestyle choices. In a recent randomized clinical trial conducted by our lab group, we examined the effects of a healthy and entirely plant-based (vegan) or a healthy omnivorous diet on cardiometabolic risk factors during an 8-week dietary intervention among 22 pairs of generally healthy identical twins.^33^ By studying identical twins, we were able to control for genetic factors and limit environmental influences, since the twins grew up in the same households and reported similar lifestyles. Both diets were replete with vegetables, legumes, fruits and whole grains and encouraged limited intake of sugars and refined starches. The vegan diet was entirely plant-based with no meat or animal products such as eggs or milk. The omnivore diet emphasized vegetables, fruits, and whole grains while limiting added sugars and refined grains and included chicken, fish, eggs, cheese, dairy, and other animal-sourced foods. The study consisted of two 4-week phases: delivered meals and self-provided meals. During the first four weeks, a meal service delivered 21 meals per week at no cost to participants—7 breakfasts, lunches, and dinners. It was expected that after 4 weeks of delivered food and counseling by health educators, the study participants would have a solid understanding of the amounts and types of foods they should purchase and prepare independently to achieve maximum adherence to the study diets in the remaining four weeks of the study. The study diet design did not include a prescribed energy restriction and was not intended to be a weight loss study. Participants were told to eat until they were satiated throughout the study.

At 3 time points—the beginning of the trial, 4 weeks, and 8 weeks—researchers weighed the participants and drew blood. The primary outcome was the difference from baseline to 8 weeks in low-density lipoprotein cholesterol (LDL-C) levels between the diet groups. Secondary outcomes included differences from baseline to 8 weeks in body weight and levels of fasting triglycerides, high-density lipoprotein cholesterol, fasting glucose, insulin, trimethylamine N-oxide (TMAO), and serum vitamin B12. The average baseline LDL-C level for the vegan-diet participants was 110.7 mg/dL and 118.5 mg/dL for the omnivore-diet participants. At the end of the study (8-week), LDL-C had dropped to 95.5 mg/dL for vegan-diet participants and 116.1 mg/dL for omnivore-diet participants. The vegan diet participants also showed an approximate 20% drop in fasting insulin and lost an average of 4.2 pounds of body weight more than the omnivore-diet participants, both statistically significant changes. Other notable findings were that vegan-diet participants had lower protein intake as a percentage of calories, lower dietary satisfaction, lower intake of vitamin B12. However, vegan-diet participants also had higher intake of vegetable servings, higher intake of dietary iron, and lower intake of dietary cholesterol. We note that the results of this study should be considered within the context of its limitations which are extensively discussed elsewhere.^33^ Our findings corroborate a previous finding showing that eating a vegan diet can improve cardiovascular health.^34^ Additional findings (i.e., microbial diversity, inflammatory markers, epigenetics) from this study of identical twins will be published in forthcoming manuscripts.

## Plant-Based Diets and the Microbiome

The gut microbiome represents another novel pathway through which a healthful plant-based diet may influence cardiovascular risk and overall health.^35,36^ The term gut microbiome refers to the complex community of microorganisms that reside in our gut. As we consume and digest food, the microbes that reside in our gut play a role. These microbes metabolize otherwise indigestible dietary substrates, such as dietary fiber, that potentially influence the cardiovascular health of the human host. High-fiber foods provide indigestible carbohydrates that feed the gut microbes, increasing microbiome diversity, which is thought to be beneficial.^37^ Fermented plant foods modulate the gut microbiome and their host’s immune system by increasing microbiome diversity and decreasing markers of inflammation.^38^

Diets high in animal-based diets have been associated with unhealthy gut microbiomes linked with chronic diseases like type 2 diabetes, obesity, and dyslipidemia. A specific example of this is the trimethylamine N-oxide (TMAO) pathway. Choline and l-carnitine, compounds derived mainly from animal foods such as red meat, poultry, and fish are used by gut microbes to generate trimethylamine (TMA), which is converted to TMAO in the liver. TMAO has been associated with a higher risk of cardiovascular events independent of traditional risk factors, and it is postulated that it adversely influences cardiac health through its effects on cholesterol and sterol metabolism, both inflammation pathways.^39^

## Study with Appetizing Plantfoods - Meat Eating Alternatives Trial (SWAP-MEAT)

In another one of our trials, Study with Appetizing Plantfoods - Meat Eating Alternatives Trial (SWAP-MEAT), participants ate at least two or more daily servings of plant-based meats for 8 weeks (plant-based phase) followed by two or more daily servings of animal meats for 8 weeks (animal phase), or vice versa.^40^ Other than eating the study food products, participants were instructed to keep all dietary habits as consistent as possible between the two phases, such that the only dietary changes were the consumption of the plant-based meat products or the animal-based meat products. We found that a plant-based diet improved several cardiovascular disease risk factors, decreasing LDL-C and TMAO.^40^ A subsequent analysis of the SWAP-MEAT trial found that biomarkers of inflammation did not improve during the plant-based phase.^41^

In SWAP-MEAT, we also observed a diet order effect of particular interest. Among the group eating an animal-based diet first and plant-based diet second, the observed mean increase in TMAO during the animal phase decreased back to baseline concentrations within 2 weeks during the plant phase, and remained stable through 8 weeks, suggesting no carryover effect. In contrast, those assigned to the plant phase first had no mean increase in TMAO, and no apparent effect to carry over, yet this group showed no mean increase in TMAO when shifted to the animal phase.^40^ Koeth et al.^42^ investigated the effect on TMAO on feeding l-carnitine, abundant in red meat, to vegetarians and reported no increase in TMAO. They speculated that this may be due to the limited ability of their “vegetarian” microbiomes to generate TMA. This finding suggests that those in the SWAP-MEAT study who were assigned to a plant-based diet for the first 8 weeks may have differentially altered their microbiomes and/or the compounds these microbes emit, compared with those who consumed an animal-based diet first, which prevented the production of TMAO in the following animal-based diet phase. We are currently investigating the potential cause of this observed order effect.

## Movement and Resistance towards Plant-Based Diets

Recently in the United States, there has been a notable increase in the adoption of plant-based diets, reflecting a trend towards more health conscious and sustainable food choices. According to recent surveys,^43^ Americans who identify as vegetarian or vegan make up at least 6 percent of the population, possibly even 10%.^44^ An individual’s decision to adopt a plant-based diet is influenced by a diverse array of motivating factors, priorities, and values.^2^ For some, the primary incentive is the compelling health benefits associated with plant-based eating. Others may be driven by environmental or ethical concerns, recognizing the ecological footprint of traditional animal agriculture and choosing plant-based diets as a more sustainable and ethical option. The alignment of plant-based dietary patterns with economic sustainability can also be an attractive aspect, as plant-based foods often offer cost-effective and accessible alternatives to animal products. However, despite the potential benefits of plant-based diets, most individuals are reluctant or resistant to change their usual dietary behaviors or unable to sustain a new diet change over time.^44,45^

Dietary choices and behaviors are complex and are determined by different individual level factors (e.g., food preference and knowledge), cultural factors (e.g., social support and social norms), economic factors like a person’s income, and the nutritional environment such as availability and access.^46,47^ Beyond the variety of reasons that a person might decide to follow a plant-based diet there are also several challenges that consumers often cite.^45,48,49^ The first is that meat has been in our diet for centuries, has a strong cultural and gastronomic significance, and may even play a role in our social identity.^50-52^ Vegetarian and vegan diets have been regarded as inconvenient as new foods or recipes may be difficult to cook or prepare, their ingredients may not always be available in stores, or satiating meals may not be available in desired restaurants.^48,53,54^ Additionally, consumers may be reluctant to even try a plant-based meal because of food neophobia.^55^

## Nutrient Considerations

For individuals who prefer not to consume certain or most animal foods, healthful and well-planned plant-based meals can provide adequate nutrition. However, without a well-planned diet, certain nutrients may be challenging to get enough from plant foods alone. These include vitamin B_12_, iron, calcium, vitamin D, omega-3 fatty acids, and zinc.^4,8,56,57^ For example, vitamin B_12_, primarily found in animal products, may necessitate supplementation or fortified foods for those following strict plant-based diets.^8,57^ With planning, individuals can navigate these nutrient considerations on a plant-based diet. A plant-based dietary pattern may also be unhealthy if it is lacking in limit specific nutrients such as vitamin B12, iron, and calcium or includes in excess food high in added sugars, refined grains, saturated fat.^58,59^ Consulting with a Registered Dietitian Nutritionist (RDs or RDNs) or a health care professional can provide personalized guidance to address individual nutritional needs.

With few exceptions all foods contain protein, yet protein is often cited as a nutrient of concern for individuals following a strict plant-based diet. Among both health professionals and the general population, it is widely believed that certain plant foods are devoid of specific amino acids and, thus, that protein adequacy cannot be supported by plant foods alone.^60^ While the proportion and quality of protein differ somewhat from one food to another, all plant foods have all 20 amino acids.^60-62^ Within a typical day of eating a variety of plant-based foods, adequate amounts of both essential and nonessential amino acids can be obtained, almost regardless of the presence or absence of animal foods.^60-62^ Additionally, it was previously thought that plant proteins with complementary amino acid profiles should be combined within each meal (e.g., consuming grains and beans/legumes together) to ensure adequate supply of all essential amino acids. However, evidence suggests that this is not necessary with a plant-based dietary pattern comprised of a wide variety of foods.^61,62^

Among recreational and professional athletes, there have been common concerns about not getting enough protein and/or calories to build strength, recover, and perform at a high level when consuming a plant-based dietary pattern.^63^ In a recent crossover pilot study,^64^ we studied the endurance and muscular strength of 24 young adult recreational athletes (12 recreational runners and 12 resistance trainers) consuming 2 predominately plant-based diets: whole-food plant-based and plant-based meat alternatives vs an omnivorous diet that favored red meat and poultry, on participant’s endurance and muscular strength. In random order, participants were randomly assigned to the 3 diets for 4 weeks each. We found that participants were able to maintain athletic performance on both an omnivorous diet and the 2 plant-based diets. These findings also mirror prior studies that observed no significant differences in athletic performance between plant-based and omnivorous diets.^65,66^ Taken together, these findings suggest that there should be no concern about receiving sufficient protein and/or calories to support athletic or recreational performance when consuming a well-planned plant-based diet.

## Plant-Based Alternatives Are Changing the Grocery Landscape

Within recent years, the global market of plant-based foods has increased dramatically, nearly 500% from 4 million in 2014 to 19.6 million in 2017.^45^ In 2021, plant-based food sales grew 6% to reach over $7.4 billion.^67^ Plant-based alternatives are specifically designed to mimic the taste and experience of eating animal-based products (e.g., dairy, cheese, meat), while being marketed as a way to accelerate the shift away from those same animal-based products. One of the most well-known types of plant-based alternatives are meat alternatives which come as burgers, sausages and hot dogs, and patties. Many plant-based meat alternative companies aim for “biomimicry” where products are virtually indistinguishable in taste, texture, and even appearance from conventional animal-based meats. Globally, the plant-based meat alternative market is projected to increase from $1.6 billion in 2019 to $3.5 billion by 2026.^68^ At the grocery store, consumers are also exposed to a large variety of plant-based milk alternatives. While such plant-based milk alternatives have been around for years (e.g., soy milk) and have grown in popularity, others have been introduced only recently (e.g., oat milk). In 2021, plant-based milks made up the largest percentage of plant-based food dollar sales.^67^ The marketplace is also expanding with new alternative products for cheese, eggs, fish, and condiments.^45^

These modern plant-based alternative products may serve as “gateway foods” helping consumers to overcome aversion and misconceptions about plant-based proteins and ultimately help them to gradually transition to healthier, plant-based and vegetarian diets.^59,69^ However, these plant-based alternative products have been scrutinized as to whether they can truly be a part of a healthful diet because they are often highly processed.^70^ Ultra-processed foods are more energy-dense and have been associated with weight gain and increased risk for the development of chronic diseases in children and adults.^71,72^ With a general shift away from animal-based proteins and an increasing demand for plant-based alternatives, more research is needed on the long-term health implications of such a dietary shift.

## Plant-Based Diets in Clinical Practice

In clinical practice, there are several approaches that aid in increasing both acceptance and adherence to a plant-based diet. Incorporating plant-based diets into clinical nutrition practice requires a patient-centered approach. It begins with understanding each patient’s unique preferences, cultural background, and current eating habits. One key strategy is to encourage simple swaps that make transitioning to a plant-based diet more accessible. This might involve replacing animal-based proteins with plant-based alternatives, such as tofu or legumes, or even swapping out half of the meat in a recipe for a plant-based alternative such as replacing half of the ground meat in a meat pasta sauce with lentils. Yet such change takes time, and therefore, clinicians should slowly expose patients to new plant-based foods and recipes, gradually expanding their culinary horizons, and slowly increase patient’s daily dietary fiber intake as patients adjust to a higher fiber diet to limit uncomfortable gastrointestinal symptoms such as bloating, constipation, and gas. This patient-centered, step-by-step approach can aid in breaking down the barriers to eating more plants. Patients don’t need to go completely vegan to see health benefits. Any increase in plant-based foods compared to their current diet increases health benefits along the spectrum. Through this approach, patients should be empowered to make sustainable, long-term changes that promote better health and well-being.

Within the in-patient setting, many hospitals are beginning to offer more plant-based foods as part of their in-patient and café menus. The University of Florida offers a plant-based menu in addition to an educational packet addressing the role of plant-based diets in chronic disease treatment and prevention.^73^ Other examples include the University of California San Francisco and Stanford Health care; both offer menus with several plant-based dishes, including alternative meat options and whole plant-based foods. We hope to see this trend continue in hospitals to both increase availability of plant-based foods for patients, but also to expose patients and health care workers to new plant-based dishes and healthy dining options.

In some situations, it may be appropriate to suggest plant-based meal delivery services to patients. These services can be cost prohibitive, and therefore may not be an appropriate recommendation for all patients. However, if patients ask, these services offer convenient and nutrient-dense options. A meal delivery service can help expose patients to new plant-based foods they are not used to cooking and provide a strong launching point into their own plant-based cooking journey through exposure to new plant foods and recipes.

When discussing plant-based diets with patients, referrals to RDNs specializing in plant-based nutrition can provide patients with expert guidance tailored to their specific needs. Implementing in-patient plant foods to patients may involve menu adjustments to ensure that plant-based options are readily available. Out-patient nutrition counseling is important to aid in patients’ adherence and enjoyment of a healthy plant-based diet, ultimately promoting better health outcomes for patients.

## Conclusion

Adopting a plant-based diet is increasingly recommended by guidelines due to their potential to improve health and environmental sustainability. Recent RCTs have shown that plant-based diets can positively impact traditional health markers and emerging chronic disease risk factors. When approached using a patient-centered counseling style and tiered exposure, patients are often receptive to these diets. To successfully incorporate plant-based diets into clinical practice, it’s crucial to meet patients where they currently are in their dietary journey, encouraging simple swaps and slowly exposing them to new foods. Additionally, plant-based meal delivery services can be a convenient option for patients, and referrals to RDNs who specialize in plant-based nutrition can provide expert guidance. Moreover, hospitals are beginning to offer more plant-based options in their menus, contributing to the broader implementation of plant-based diets within health care systems. Overall, these strategies can empower patients to make sustainable changes that promote better health and well-being, aligning with the recommendations of professional and medical organizations endorsing plant-based diets.
